# Management of Post-Dural Puncture Headache in Postpartum Women: A Clinical and Therapeutic Update

**DOI:** 10.7759/cureus.107980

**Published:** 2026-04-29

**Authors:** Pablo R Durán Monge, Eimy Zúñiga Gómez, Laura C Castro Rivero

**Affiliations:** 1 Critical Care Crew, Soporte Asistido Aéreo y Terrestre, San José, CRI; 2 Gynecology, Hospital San Juan de Dios, San José , CRI; 3 Gynecology, Hospital San Juan de Dios, San José, CRI

**Keywords:** cerebrospinal fluid leak, epidural anesthesia, epidural blood patch, orthostatic headache, post-dural puncture headache, postpartum period

## Abstract

Post-dural puncture headache (PDPH) is a common complication of neuraxial anesthesia, particularly in the obstetric population, where its incidence is higher due to the frequent use of epidural techniques and the occurrence of accidental dural puncture. The condition arises from cerebrospinal fluid leakage following disruption of the dura mater, leading to intracranial hypotension, compensatory cerebral vasodilation, and traction of pain-sensitive structures, which collectively explain its characteristic orthostatic presentation. Pregnancy-related physiological changes, including increased intra-abdominal pressure and epidural venous engorgement, further contribute to its development and severity.

Clinically, PDPH presents as a positional headache that worsens in the upright position and improves with recumbency, often accompanied by nausea, photophobia, diplopia, and auditory symptoms. Diagnosis is primarily clinical and relies on a history of recent neuraxial procedures and the absence of red flag neurological signs. However, careful evaluation is necessary to exclude other serious postpartum conditions such as preeclampsia, cerebral venous sinus thrombosis, and intracranial hemorrhage.

Prevention focuses on optimizing procedural techniques, including the use of small-gauge atraumatic needles, minimizing puncture attempts, and ensuring proper training. Management strategies depend on symptom severity and range from conservative measures, such as hydration, analgesics, and caffeine, to pharmacological interventions. In moderate-to-severe cases, the epidural blood patch remains the gold standard treatment, offering high success rates. Given its potential impact on maternal function, breastfeeding, and mental health, a multidisciplinary and patient-centered approach is essential to improve outcomes and reduce long-term complications.

## Introduction and background

Post-dural puncture headache (PDPH) has been recognized as a complication of neuraxial procedures since the 1950s, when early reports described persistent and long-lasting headaches in affected patients. In the context of obstetric anesthesia, accidental dural puncture (ADP) remains a clinically relevant event, with an estimated incidence ranging from 0.7% to 1.5%, of which approximately 60% to 80% progress to severe PDPH [1]. Over time, the understanding of this condition has evolved considerably, with increasing recognition of its potential to extend beyond the acute phase and contribute to chronic headaches and other long-term sequelae [2].

The incidence of PDPH varies according to the type of neuraxial procedure performed, with higher rates observed following lumbar puncture and epidural anesthesia compared to other techniques [3]. In postpartum women, this condition represents a significant clinical burden, as it can interfere with essential daily activities such as ambulation, breastfeeding, and effective maternal-infant interaction. Furthermore, the persistence of symptoms is not uncommon, as chronic headaches may develop and last for several months in up to 30% of women who experience ADP with the use of large-bore needles [2]. 

Given these implications, the postpartum period emerges as a particularly sensitive window in which optimal maternal recovery and early bonding with the newborn are essential. The presence of PDPH during this stage can significantly compromise quality of life and may increase healthcare utilization due to prolonged symptomatology and the need for interventions such as the epidural blood patch (EBP) [4]. Consequently, a focused and tailored approach in obstetric populations is warranted, aiming not only to prevent the occurrence of PDPH but also to ensure timely and effective management to reduce the risk of long-term complications [5].

Several risk factors for the development of PDPH have been consistently identified, including younger age, female sex, and the use of larger gauge needles [3]. These factors, combined with procedural elements, underscore the importance of preventive strategies in clinical practice. Among these, careful technique during epidural placement and appropriate selection of needle type have been shown to play a crucial role in reducing the incidence of PDPH [2]. Although additional prophylactic measures, such as the administration of cosyntropin, have been explored, current evidence suggests limited efficacy in significantly decreasing the occurrence of this complication [6].

The objective of this article is to analyze PDPH in postpartum women, emphasizing its clinical significance in obstetric anesthesia, associated risk factors, and impact on maternal recovery, while reviewing current prevention strategies and therapeutic approaches to optimize outcomes and reduce potential complications.

## Review

Methodology

This study was developed as a structured narrative review aimed at providing an updated and clinically integrated analysis of PDPH in postpartum women, with particular emphasis on its epidemiology, risk factors, clinical presentation, and contemporary prevention and management strategies. The review was conducted in accordance with the Scale for the Assessment of Narrative Review Articles (SANRA) framework and followed a predefined methodological protocol established before literature screening [7]. Given the variability in clinical presentation, procedural factors, and therapeutic approaches in obstetric populations, a narrative interpretative synthesis was selected over quantitative pooling in order to integrate physiological, anesthetic, and postpartum considerations into a coherent and clinically applicable framework [7]. Special attention was given to the impact of ADP, the role of needle characteristics and procedural technique, and the effectiveness of both conservative and interventional treatments, including EBP. The objective was to provide a structured synthesis capable of supporting clinical decision-making in obstetric anesthesia and postpartum care.

A comprehensive literature search was conducted in PubMed, Scopus, and Web of Science, including peer-reviewed articles published in English or Spanish between January 2020 and December 2026. The final search was performed in March 2026. This timeframe was selected to capture contemporary advances in obstetric anesthesia techniques, preventive strategies, and emerging therapeutic interventions for PDPH. Foundational studies were incorporated when necessary to contextualize pathophysiological mechanisms and the historical evolution of management strategies. The search strategy combined Medical Subject Headings and free-text terms using Boolean operators related to PDPH, postpartum headache, obstetric anesthesia, epidural anesthesia, spinal anesthesia, ADP, EBP, cerebrospinal fluid leak, and neuraxial complications. Searches were conducted in titles and abstracts as well as indexed subject headings to maximize sensitivity. No references were used in the methodology section, as it reflects the authors’ procedural approach.

The study selection process followed a structured approach consistent with PRISMA recommendations. The initial search yielded 113 records [8]. After removal of duplicates, 86 articles remained for title and abstract screening. Of these, 52 underwent full-text evaluation, and 21 studies were included in the final synthesis. Selection was performed independently by two authors, with disagreements resolved through discussion and consensus. Inclusion criteria comprised studies involving postpartum women undergoing neuraxial anesthesia that reported on the incidence, risk factors, clinical presentation, prevention, or management of PDPH. Eligible study designs included randomized controlled trials, observational studies, systematic reviews, meta-analyses, expert consensus statements, and international clinical guidelines. Exclusion criteria included non-peer-reviewed publications, isolated case reports, editorials without clinical outcome data, purely technical descriptions without relevance to postpartum populations, redundant datasets, and studies not directly addressing the defined outcomes.

The primary outcome of interest was the resolution of PDPH, while secondary outcomes included the need for EBP, recurrence rates, treatment-related complications, and functional impact on postpartum recovery. Data extraction was performed independently by two authors using a predefined framework that included study design, patient population, type of neuraxial procedure, incidence of ADP, clinical characteristics of headache, management strategies, timing and success of EBP, and reported maternal outcomes.

Methodological quality and internal validity were assessed narratively based on predefined criteria, including risk of bias, sample size, follow-up duration, consistency of outcome definitions, and reproducibility of findings. Although no formal scoring system was applied, greater interpretative weight was assigned to higher-level evidence and guideline-supported recommendations. Potential sources of bias, including publication bias, selection bias, and heterogeneity in study populations and interventions, were considered during the synthesis and interpretation of results.

Reference lists of included studies were manually screened to identify additional relevant publications. Given its narrative design, this review is subject to inherent limitations, including potential selection bias and the absence of pooled quantitative estimates. Artificial intelligence-based tools, specifically ChatGPT (OpenAI), were used solely to support literature organization and enhance the structural coherence of the manuscript. All processes involving critical appraisal, data synthesis, and final interpretation were conducted independently by the authors to ensure methodological rigor and scientific validity.

Anatomy, physiology, and pathophysiology

The meninges are composed of three distinct layers: the dura mater, arachnoid mater, and pia mater. The dura mater, which represents the outermost and most fibrous layer, is of particular clinical relevance in neuraxial procedures, as it is the structure most punctured during epidural or spinal anesthesia. Disruption of this layer may lead to cerebrospinal fluid leakage and the subsequent development of PDPH [2,9]. Anatomically, the epidural space is located external to the dura mater, whereas the subarachnoid space lies between the arachnoid and pia mater and contains circulating cerebrospinal fluid. PDPH typically arises when a breach in the dura mater permits the leakage of cerebrospinal fluid from the subarachnoid space into the epidural space, thereby altering normal pressure dynamics [2]. In addition, the meninges contain vascular and neural structures that are sensitive to changes in intracranial pressure, which contribute to the development of headache symptoms in affected patients [9].

Cerebrospinal fluid plays a fundamental role in maintaining intracranial homeostasis. It is produced by the choroid plexus and subsequently absorbed into the venous system, circulating within the subarachnoid space to provide both mechanical protection and metabolic support to the brain and spinal cord. Under normal conditions, this dynamic equilibrium ensures stable intracranial pressure. However, disruption of the dura mater can lead to continuous cerebrospinal fluid loss, resulting in a reduction in both volume and pressure. This imbalance is a central mechanism in the development of PDPH, as intracranial hypotension directly contributes to symptom generation [9].

Physiological changes associated with pregnancy further influence the development and severity of PDPH. Increased intra-abdominal pressure during pregnancy may exacerbate cerebrospinal fluid leakage, thereby intensifying the effects of intracranial hypotension. Additionally, hormonal adaptations contribute to epidural venous engorgement, which may increase the likelihood of dural puncture during neuraxial procedures. These hormonal fluctuations also affect vascular tone, potentially influencing both the intensity and duration of headache symptoms in the postpartum period [2].

From a pathophysiological perspective, the persistence of cerebrospinal fluid leakage through a dural defect leads to a sustained decrease in intracranial pressure, resulting in intracranial hypotension. In response to this reduction in cerebrospinal fluid volume, compensatory cerebral vasodilation occurs in accordance with the Monro-Kellie doctrine, further contributing to headache development. Moreover, the decrease in cerebrospinal fluid pressure can cause a downward displacement of brain structures, generating traction on pain-sensitive meningeal and vascular elements. This mechanism explains the characteristic orthostatic nature of the headache, in which symptoms worsen in the upright position and improve with recumbency [1,2].

Epidemiology and risk factors

The incidence of PDPH varies depending on the population and the type of neuraxial procedure performed. In the general population undergoing neuraxial techniques, the reported incidence ranges from approximately 0.38% to 6.3% [10]. However, in the obstetric population, the incidence is notably higher, primarily due to the increased frequency of epidural anesthesia. ADP occurs in approximately 0.7% to 1.5% of obstetric cases, and among these, 60% to 80% of patients subsequently develop PDPH [1]. 

ADP represents one of the most significant risk factors for the development of PDPH. The risk is markedly elevated in these cases, with nearly 70% to 80% of affected women reporting clinically significant headaches [2]. Furthermore, unintentional dural puncture has been consistently identified as a high-risk event not only for acute symptom development but also for the persistence of chronic headache syndromes [11].

Several patient-related factors have been associated with an increased likelihood of developing PDPH. Younger age has been identified as a relevant risk factor, as increased dural elasticity in younger individuals may predispose them to a higher incidence of dural puncture. In addition, female sex, particularly within the obstetric population, is associated with a greater susceptibility to this condition, possibly due to hormonal and anatomical influences [3]. A lower body mass index has also been linked to an increased risk, although evidence specifically addressing this association in obstetric patients remains limited [4]. Moreover, a prior history of PDPH or chronic headache disorders further increases the likelihood of recurrence following subsequent dural puncture [12].

Obstetric-specific factors also contribute to the development of PDPH. Prolonged labor has been associated with an increased risk, potentially due to the cumulative physical stress experienced during delivery. Additionally, increased intra-abdominal pressure during labor may exacerbate the likelihood of dural puncture and subsequent cerebrospinal fluid leakage. Dehydration during labor has also been proposed as a contributing factor, as it may influence cerebrospinal fluid dynamics and predispose patients to symptom development [4].

Procedure-related factors play a critical role in determining the risk of PDPH. The size and type of needle used are among the most important determinants, with larger gauge and cutting needles, such as Quincke needles, being associated with a higher incidence compared to smaller gauge and atraumatic needles, including Whitacre and Sprotte designs [3]. In addition, an increased number of puncture attempts has been shown to correlate with a higher likelihood of headache development. The orientation of the needle bevel also influences risk, as insertion parallel to dural fibers may reduce the probability of dural disruption. The experience and technical skill of the practitioner performing the procedure are key determinants, with less experienced operators being associated with a higher incidence of ADP and subsequent PDPH [4].

Clinical presentation

PDPH is primarily characterized by an orthostatic headache, which worsens when the patient assumes an upright position and improves upon lying down. This positional component represents a key diagnostic feature and is central to the clinical identification of the condition. The headache typically presents in the frontal, occipital, or generalized regions of the head and may vary in distribution among patients [2,11]. In terms of intensity, the pain can range from mild to severe and is often sufficiently disabling to interfere with daily activities, including basic functional tasks in the postpartum period. The quality of the headache is commonly described as throbbing or pressure-like, contributing to significant patient discomfort and distress [11,13].

In addition to the core symptom, a range of associated manifestations may be present. Gastrointestinal symptoms such as nausea and vomiting frequently accompany the headache and can exacerbate the overall clinical burden. Neurological symptoms are also notable, including photophobia and diplopia, the latter often related to involvement of the sixth cranial nerve. Auditory symptoms, although less common, may include tinnitus and hypoacusis, further reflecting the widespread effects of intracranial pressure changes [11,14].

The temporal profile of PDPH is relatively well defined. Symptom onset typically occurs within 24 to 72 hours following the dural puncture procedure. If left untreated, the headache may persist for several days to weeks, significantly impairing quality of life, particularly in postpartum women who require adequate functional capacity for newborn care. Although traditionally considered a self-limiting condition, emerging evidence suggests that PDPH may not always resolve completely. A proportion of affected women may develop chronic headache syndromes that persist for months or even years after the initial episode, highlighting the potential for long-term consequences and the importance of timely recognition and management [2,11,13].

Diagnosis and differential diagnosis

The clinical diagnosis of PDPH is primarily based on patient history and characteristic symptomatology. A recent history of a neuraxial procedure, such as epidural or spinal anesthesia, is a fundamental element in establishing the diagnosis, particularly when a dural puncture has occurred either intentionally or unintentionally. The incidence of PDPH is significantly higher in cases of ADP, with up to 80% of affected women developing symptoms. This temporal association between the procedure and symptom onset is essential for clinical recognition [12,14].

A defining feature of PDPH is its positional nature. The headache typically worsens when the patient is in an upright position and improves upon lying down, reflecting the underlying pathophysiology related to cerebrospinal fluid leakage and intracranial hypotension. This orthostatic characteristic serves as a key diagnostic criterion and helps distinguish PDPH from other types of headaches [11]. In addition, the absence of red flag neurological signs supports the diagnosis, as patients with PDPH generally do not present with seizures, focal neurological deficits, or altered mental status [13].

The presence of atypical features should prompt consideration of alternative diagnoses. A headache that does not vary with position raises suspicion for other etiologies, while the occurrence of seizures or focal neurological deficits may indicate more serious conditions such as cerebral venous sinus thrombosis or intracranial hemorrhage. Similarly, altered mental status is a critical warning sign that necessitates further evaluation to exclude other intracranial or systemic pathologies [13].

In postpartum patients, the differential diagnosis of headache is broad and requires careful clinical assessment. Conditions such as preeclampsia and eclampsia must be considered, particularly given their potential severity and association with hypertensive disorders of pregnancy. Cerebral venous sinus thrombosis represents another important differential diagnosis, as it can present with headache but is often accompanied by additional neurological symptoms. Other critical conditions include subarachnoid or intracranial hemorrhage, which should be ruled out in cases of severe or atypical headache presentation. Infectious processes such as meningitis or encephalitis may also present with headache and altered mental status, requiring prompt differentiation. Additionally, primary headache disorders, including migraine and tension-type headache, may coexist with PDPH and complicate the clinical picture [13].

Diagnostic testing is not routinely required in typical cases but becomes essential when the presentation is atypical, severe, or refractory to initial management. Neuroimaging, particularly computed tomography or magnetic resonance imaging, is indicated to exclude other intracranial pathologies. Magnetic resonance imaging may also reveal characteristic findings of PDPH, such as pachymeningeal enhancement, which can support the diagnosis [15]. Laboratory investigations are guided by the suspected alternative diagnosis and may include blood tests in cases of suspected preeclampsia or cerebrospinal fluid analysis when an infectious etiology is considered [13]. 

Prevention

Preventive strategies for PDPH are centered on optimizing procedural techniques, strengthening institutional practices, and ensuring early recognition of complications. From a technical perspective, the use of small-gauge, atraumatic needles represents one of the most effective measures to reduce the incidence of PDPH. These needles, particularly pencil-point designs, produce less dural trauma and consequently minimize cerebrospinal fluid leakage when compared to conventional cutting needles, resulting in a lower risk of headache development [16]. In addition, proper needle orientation during insertion plays a critical role, as aligning the bevel parallel to dural fibers can decrease the likelihood of dural tearing and further reduce the risk of PDPH [5].

Equally important is the limitation of puncture attempts during neuraxial procedures. Multiple attempts are associated with an increased probability of ADP, which significantly elevates the risk of subsequent headache. Therefore, minimizing the number of attempts through careful planning and technique is essential. In situations where anatomical landmarks are difficult to identify, the use of ultrasound guidance has been shown to improve the accuracy of needle placement. This approach enhances procedural success and reduces the likelihood of inadvertent dural puncture, thereby contributing to prevention [5].

At the institutional level, structured training and supervision of clinicians performing neuraxial procedures are fundamental components of prevention. Adequate training improves technical proficiency and consistency, which directly impacts complication rates, including PDPH. The implementation of standardized protocols for neuraxial anesthesia further reinforces best practices and ensures uniformity in clinical approach, thereby reducing variability and enhancing patient safety [5].

Early recognition also plays a crucial role in mitigating the severity and progression of PDPH. Prompt identification of ADP allows for immediate interventions, such as the placement of an intrathecal catheter, which may reduce the incidence or severity of subsequent symptoms [17]. Furthermore, close monitoring of postpartum women who have undergone neuraxial procedures facilitates early detection of headache symptoms and timely initiation of management strategies. This approach is particularly important in preventing the progression to chronic headache syndromes and minimizing the overall clinical impact of the condition [1,11].

Therapeutic management

The management of PDPH encompasses a range of therapeutic strategies that vary according to symptom severity, functional impact, and response to initial treatment. Conservative management is typically considered the first-line approach in mild cases and aims to provide symptomatic relief while allowing for spontaneous resolution. Adequate hydration, administered either orally or intravenously, is commonly recommended as a supportive measure, although its direct effect on cerebrospinal fluid dynamics remains limited. Analgesics, including acetaminophen and nonsteroidal anti-inflammatory drugs, are frequently used to control pain and improve patient comfort. In addition, caffeine may be administered orally or intravenously, as it promotes cerebral vasoconstriction, thereby counteracting the vasodilation associated with the pathophysiology of PDPH. Patients are also advised to maintain relative rest, avoiding strict immobilization, which has not been shown to provide additional benefit and may, in some cases, worsen symptoms [5,18].

When conservative measures are insufficient, pharmacological interventions may be considered. Theophylline, an adenosine receptor antagonist, has been proposed as a therapeutic option due to its effects on cerebral blood flow regulation. Gabapentin has also been explored for its neuromodulatory properties and potential benefit in symptom control, although current evidence supporting its use remains limited. Similarly, the use of triptans is supported by limited data, and the role of corticosteroids in the management of PDPH continues to be controversial, with inconsistent evidence regarding their efficacy [18].

In cases of moderate to severe headache or when conservative therapy fails to provide relief within 24 to 48 hours, the EBP is considered the gold standard treatment. This intervention is also indicated when there is significant functional impairment. The procedure involves the injection of approximately 15 to 20 mL of autologous blood into the epidural space, typically at or near the level of the original dural puncture. The therapeutic effect is achieved through multiple mechanisms, including an immediate tamponade effect, sealing of the dural defect, and restoration of cerebrospinal fluid pressure. The reported success rate of the initial procedure ranges from 70% to 90%, although some patients may require a repeat intervention to achieve complete symptom resolution. While generally safe, potential complications include localized back pain, infection, and, in rare cases, neurological complications [8,19].

Alternative interventions have also been investigated, particularly in patients who are not candidates for EBP or in whom the procedure is unsuccessful. The sphenopalatine ganglion block has emerged as a minimally invasive option with potential benefits in symptom relief [18]. Additionally, epidural saline infusion and experimental approaches such as fibrin glue patches are being explored for their potential role in both the prevention and treatment of PDPH, although current evidence remains limited and further research is required to establish their effectiveness [20].

Postpartum-specific considerations

The management of PDPH in postpartum women requires careful consideration of breastfeeding safety, functional limitations, psychological impact, and the need for coordinated multidisciplinary care. With regard to pharmacological treatment, several commonly used medications are considered compatible with breastfeeding. Acetaminophen and nonsteroidal anti-inflammatory drugs are generally regarded as safe, with acetaminophen demonstrating a relatively high milk-to-plasma concentration ratio, reflecting significant transfer into breast milk without clinically relevant adverse effects in most cases [21]. Caffeine is also considered safe when used in moderate doses; however, caution is warranted with other pharmacological agents due to the potential for neonatal exposure and associated risks. Despite the established safety of many medications, breastfeeding discontinuation remains a concern, often driven by inconsistent or overly cautious recommendations from healthcare providers rather than clear evidence of harm [22].

Beyond pharmacological considerations, PDPH can have a significant functional impact on postpartum women. The characteristic orthostatic nature of the headache may substantially limit maternal mobility, impairing the ability to perform routine activities and hindering adequate recovery. These limitations can directly affect a mother’s capacity to care for her newborn, potentially interfering with essential activities such as feeding, holding, and bonding, thereby impacting both maternal and infant well-being [4,10].

The psychological burden associated with PDPH should also be recognized. Persistent pain can exacerbate fatigue and stress during a period already characterized by significant physical and emotional demands [2]. Furthermore, the condition may negatively influence postpartum mental health, increasing the risk of anxiety and depressive symptoms, which are already prevalent during this stage. These factors highlight the importance of addressing not only the physical but also the emotional dimensions of the condition [4].

Given these complexities, a multidisciplinary approach is essential for optimal management. Effective coordination between anesthesiology, obstetrics, and pediatrics ensures comprehensive care that addresses both maternal and neonatal needs. In addition, individualized, patient-centered care is crucial, as management strategies should be tailored to the specific clinical context, symptom severity, and personal circumstances of each patient to achieve the best possible outcomes [5].

## Conclusions

PDPH is fundamentally driven by cerebrospinal fluid leakage secondary to dural disruption, leading to intracranial hypotension, compensatory vasodilation, and traction of pain-sensitive structures, which together explain its characteristic orthostatic presentation and symptomatology.

The obstetric population is at increased risk of developing PDPH due to a combination of patient-related, procedural, and pregnancy-specific factors, with ADP representing the most significant determinant of both acute and potentially chronic manifestations.

Early clinical recognition based on characteristic features, along with the implementation of preventive strategies and timely, individualized management, is essential to reduce functional impairment, prevent long-term complications, and optimize maternal outcomes in the postpartum period.
